# Infection control measures in ophthalmology during the COVID-19 outbreak: A narrative review from an early experience in Italy

**DOI:** 10.1177/1120672120927865

**Published:** 2020-05-17

**Authors:** Daniele Veritti, Valentina Sarao, Francesco Bandello, Paolo Lanzetta

**Affiliations:** 1 Department of Medicine – Ophthalmology, University of Udine, Udine, Italy; 2 Department of Ophthalmology, Ospedale Santa Maria della Misericordia, Azienda Sanitaria Universitaria Friuli Centrale (ASUFC), Udine, Italy; 3 Istituto Europeo di Microchirurgia Oculare (IEMO), Udine, Italy; 4 Vita-Salute San Raffaele University, Milan, Italy; 5 IRCCS San Raffaele Scientific Institute, Milan, Italy

**Keywords:** COVID-19, coronavirus, SARS-CoV-2, triage, face mask, personal protective equipment

## Abstract

**Introduction:**

The novel coronavirus (SARS-CoV-2) is infecting people and spreading easily from person-to-person. Cases have been detected in most countries worldwide. Italy is one of the most affected countries as of 30 March 2020. Public health response includes a rapid reorganization of the Italian National Healthcare System in order to reduce transmission of COVID-19 within hospitals and healthcare facilities, while optimizing the assistance to patients with severe COVID-19 complications.

**Methods:**

We analysed the actions that were taken in three ophthalmology centres in northern Italy during the SARS-CoV-2 outbreak and how these measures affected patient’s attendance. In addition, due to the rapidly evolving scenario, we reviewed the evidence available during the course of this pandemic.

**Results:**

A full reorganization of ophthalmology services is mandatory according to current existing infection containment measures in order to continue dispensing urgent procedures without endangering the community with amplification of the diffusion chain. Ophthalmologists are considered at elevated risk of exposure when caring patients and vice versa, due to their close proximity during eye examination. High volumes of procedures typically generated by ophthalmologists with concurrent implications on the risk of infection are considered when re-assessing healthcare facilities reorganization.

**Conclusion:**

Containment measures in the event of pandemic due to infective agents should be well known by healthcare professionals and promptly applied in order to mitigate the risk of nosocomial transmission and outbreak.

## Introduction

In December 2019, an atypical pneumonia of unknown origin was first reported in a group of patients in Wuhan, China.^1^ Chinese authorities identified a new betacoronavirus (severe acute respiratory syndrome coronavirus 2 (SARS-CoV-2)) as the cause of the outbreak.^2^ SARS-CoV-2 is highly contagious and has evolved into a global health threat within weeks. As of 30 March 2020, the ongoing outbreak of coronavirus disease 2019 (COVID-19) originating in Wuhan had caused more than 770,000 confirmed cases and more than 37,000 deaths, with 178 countries/sovereignties affected worldwide.^3^ At the same date, Italy was one of the most affected countries with more than 100,000 confirmed cases and more than 11,000 deaths. Cities like Milan, Bergamo and Brescia, in Lombardy, reported an exponential growth of hospitalized people infected by SARS-CoV-2. Since 10 March 2020, Italy has been on lockdown. Schools, universities, museums and most shops have been closed and the National Healthcare System is trying to cope with the flood of patients needing hospitalization and intensive care unit support.^4^ The emergence of a novel viral pneumonia constitutes an unprecedented threat and challenge to the community as well as to the healthcare system. Strong hygiene and containment measures and individual citizen responsibility, including strict self-isolation measures, have been instituted to slow down virus transmission. Also, a rapid infection control response is essential to contain and mitigate the risk of nosocomial transmission and outbreak. In light of this, since the lockdown, we rapidly modified the organization of our ophthalmology clinics in order to stepping up control measures for SARS-CoV-2 infection. Working modalities are continuously evolving according to local regulations and available guidelines. This article aims to share the local experience of three centres in northern Italy (Department of Ophthalmology, University Vita-Salute, IRCCS Ospedale San Raffaele, Milan; Department of Ophthalmology, ASUFC, Udine; and Istituto Europeo di Microchirurgia Oculare (IEMO), Udine) in order to educate ophthalmologists on necessary measures to minimize impact on both healthcare workers and patients. Transitional adopted measures are also discussed with respect to recent published data.

## Methods

A review of relevant documents through Internet and database search was conducted in relation to SARS-CoV-2. Pertinent evidence was selected in order to implement strategies to avoid virus infection in ophthalmology. We describe the scenario of three ophthalmology centres in northern Italy during the SARS-CoV-2 outbreak. The Department of Ophthalmology of the University Vita-Salute, IRCCS Ospedale San Raffaele is located in Milan, a city with 1.3 million inhabitants and a dense and extended urban region with a total population of 5 million people which has already been hit hard by the COVID-19 outbreak. The Department of Ophthalmology of ASUFC is located in Udine, a city with 100,000 inhabitants and serves Italy’s north-easternmost region (1.2 million inhabitants). In this region, the COVID-19 epidemic curves are not yet as dramatic as in Milan and Lombardy, with a slower increase rate. IEMO is also located in Udine. It is an ambulatory surgery centre (ASC) focused on providing same-day diagnostic and surgical care, especially for retinal conditions and intravitreal therapies. We analysed the actions that were taken in the above cited centres and how these measures affected patient’s attendance. In addition, due to the rapidly evolving scenario, we reviewed the evidence available during the course of this pandemic.

## Observation

### COVID-19: the epidemic and the challenges

The SARS-CoV-2 was found to be a positive-sense single-stranded RNA virus belonging to the genus *Betacoronavirus*. Based on the observation of data, the trend in an increasing incidence largely follows exponential growth. The current estimate of the mean incubation period for the COVID-19 was 6.4 days, which ranges from 2.1 to 11.1 days.^5,6^ SARS-CoV-2 is spread by human-to-human transmission via droplets or direct contact. Several reports suggest that the virus can cause conjunctivitis and possibly be transmitted by aerosol contact with conjunctiva.^7,8^ Despite recognition that transmission occurs mostly via symptomatic individuals, there are reports of asymptomatic individuals who infected multiple family members.^9–11^ These reports underscore the need for prevention of cross-infection. Evidence related to transmissibility and mortality alert the healthcare community of the importance of vigilance, preparation, active management and protection. In high-risk sites and clinical settings, where crowding is identified as a major concern, rigour in the use of recommended precautions for all patients including public health and infection control measures is urgently needed. At the time of the latest revision of this article (22 April 2020), tens of thousands of healthcare workers have developed infection internationally, including 3400 in China and more than 15,000 in Italy, as well as thousand of infections in Americans. Latest estimates from Lombardy suggest that 20% of healthcare professionals are positive for SARS-CoV-2. To date, more than 140 doctors (including one ophthalmologist) died from COVID-19 in Italy and among the healthcare workers who died in Wuhan, three were Chinese ophthalmologists, including Dr Li Wenliang, who was probably infected while treating an asymptomatic glaucoma patient.^12,13^ It is essential to limit human-to-human transmission, to reduce secondary infections among healthcare workers and prevent transmission amplification events within hospitals and health services. Since the end of February, hospitals in northern Italy have been reorganized. Most of them have special areas for COVID-19 patients. Some hospitals are acting as hubs to collect patients with COVID-19 and related diseases. Most of the outpatient clinics have been shut down and non-urgent visits are postponed to make resources available for the most severe cases. While the situation is evolving rapidly, newer documents and guidelines are available to advice on best practice.

### Strategies to prevent transmission in ophthalmology clinics

We present a real-life scenario on reorganization of clinical activity at our clinics in order to:

1. Prevent nosocomial transmission amplification events.
2. Provide adequate care of patients with non-deferrable ocular conditions.
3. Facilitate the conversion of a routine care hospital to a hub hospital for assisting patients experiencing COVID-19 complications.

Strategies from all the three ophthalmic centres were generally consistent with some differences that will be detailed below and summarized in Table 1.

**Table 1.** Summary of recommendations.

Measures in this study (10 March 2020)	AAO recommendations (29 March 2020)	RCOphth recommendations (19 March 2020)	SOI and AIMO recommendations (18–28 March 2020)
Minimize chance for exposures			
Suspension of elective clinical services	Supported	Supported	Supported
Suspension of elective surgical procedures	Supported	Supported	Supported
Maintain intravitreal therapies	Not mentioned	Supported	Supported
Patient triage			
Safe triage and isolation of patients with symptoms of suspected COVID-19 or other respiratory infection	Supported	Supported	Supported
Reduction of droplet generation and infection transmission			
Promote cough etiquette and hand hygiene	Supported	Supported	Supported
Slit-lamp barriers	Supported	Supported	Supported
Avoid speaking during slit-lamp examination	Supported	Not mentioned	Supported
Prefer contact tonometry	Not mentioned	Not mentioned	Not mentioned
Use of personal protective equipment			
Face masks for symptomatic patients	Supported	Supported	Supported
Face masks for healthcare personnel assisting asymptomatic patients	Not routinely required	At discretion	Supported
Face masks for asymptomatic patients	Not routinely required	At discretion	Supported
Other measures			
Environmental control	Supported	Supported	Supported
Staff education	Supported	Supported	Supported
Patient education	Not mentioned	Not mentioned	Supported

AAO: American Academy of Ophthalmology; RCOphth: The Royal College of Ophthalmologists; SOI: Società Oftalmologica Italiana; AIMO: Associazione Italiana Medici Oculisti.

#### Minimize chance for exposures

Avoid hospital-related transmission of the virus. Enforce facility policies and practices to minimize exposures to SARS-CoV-2. Implement measures even before patient arrival with the aim of lowering patient attendance. We applied the following actions:

- Suspension of elective clinical services and rescheduling appointments to patients with non-urgent conditions. Due to the relevant volume of subjects attending the outpatient service, selected physicians (including residents), nurses and administrative personnel have been dedicated to screen patients’ records and to contact scheduled patients via phone. In routine conditions, 3000 patients at the hospital in Milan, 590 patients at the hospital in Udine and 110 patients at the ASC are typically scheduled for an ophthalmic evaluation or a diagnostic procedure in 1 week. Within the first week of the outbreak, a reduction in patients’ attendance by 70%–80% was attained in all institutions. Six hundred patients in Milan, 113 patients at the Udine hospital and 30 patients in the ASC were seen due to urgent ocular conditions or underwent non-deferrable diagnostic exams.
- As for surgery, cataract interventions and other elective procedures have been postponed. Intravitreal therapies were maintained to avoid any delay. There are two reasons for not deferring intravitreal injections. Any delay may possibly cause both anatomical and functional irreversible worsening. Furthermore, rescheduling high volumes of injections poses challenges on capacity and available slots. Cancellation of elective surgeries allowed to increase the interval between procedures avoiding massive presence of patients at the clinics and hopefully reducing the risk of infection. Under normal circumstances, 141 patients in Udine and 330 patients in Milan undergo surgical procedures or intravitreal injections per week. Within the first week of the Italian outbreak, after rescheduling, 71 patients at the Udine hospital and 80 in Milan underwent non-deferrable surgical procedures (retinal detachment surgery, glaucoma surgery, ocular oncology surgery, trauma) and intravitreal injections.

Scheduled intravitreal injections were mostly maintained at the ASC. A number of patients planned for intravitreal therapies at the hospital centres cancelled their appointments. This rate was higher in the Milan centre due to the subset of population referring to the San Raffaele Hospital. Those patients are often residents from other Italian regions who experienced major difficulties in travelling during the outbreak and were at high risk of quarantine after their return home. Some patients skipped the appointment because they did not perceive the urgency of the therapy.

Fortunately, the total time spent at the health facilities for receiving intravitreal therapies did change after the outbreak and lockdown. At the hospitals and ASC, respectively, the mean total time decreased from 185 to 102 min and from 1 h to 32 min.

The recently released recommendations from the American Academy of Ophthalmology (AAO) list elective/non-urgent and elective urgent procedures. Among the non-urgent procedures are: strabismus surgery, standard cataract extraction, lid surgery/blepharoplasty, cosmetic oculoplastics, intraocular lens exchange, refractive surgery, large and stable macular hole repair and stable epiretinal membrane peel. Poorly controlled glaucoma surgery, macula-off retinal detachment repair, orbital biopsy, optic nerve sheath fenestration, congenital cataract and other non-deferrable procedures are considered as elective urgent procedures. Macula threatening retinal detachment, endophthalmitis and open globe repair/full-thickness corneal laceration are considered urgent/emergent surgeries. The abovementioned recommendations state that ‘urgency’ is determined by physician judgement and must always take into account individual patient medical and social circumstances.^14^

- In order to minimize the influx of patients and caregivers, guidance is given to convert all accesses that do not strictly require a visit of the patient into a phone contact.

#### Patient triage

It is particularly important to protect individuals at increased risk for adverse outcomes from COVID-19 (e.g. older individuals with comorbid conditions). In order to protect the patients attending the clinic and the healthcare staff, a rapid safe triage and isolation of patients with symptoms of suspected COVID-19 or other respiratory infections (e.g. fever and cough) are mandatory. We implemented the following steps:

- A triage station is set up outside the facility to screen patients prior to entering the clinic.
- Physical barriers are installed (e.g. glass or plastic windows) at reception areas to limit close contact between triage personnel and potentially infectious patients.
- Visual alert icon (e.g. signs and posters) at the entrance and in strategic places (e.g. waiting areas and elevators) is posted to provide patients and healthcare professionals with instructions about hand hygiene, respiratory hygiene and cough etiquette.
- Supplies for respiratory hygiene and cough etiquette, including alcohol-based hand rub with 60%–95% alcohol, tissues and no-touch receptacles for disposal, are positioned at entrances, waiting rooms and patient check-ins.
- Access is limited only to patients with non-deferrable ocular conditions. Presence of caregivers, if not strictly necessary, is not allowed inside the clinic. When needed, only one accompanying person is permitted.
- Triage of patients with respiratory symptoms is prioritized.
- At the time of check-in, patients are asked about the presence of symptoms of a respiratory infection and history of travel to areas experiencing transmission of COVID-19 or contact with possible COVID-19 cases in the past 14 days.
- Triage personnel wear surgical face masks. These are also provided to patients with symptoms of respiratory infection at check-in. At the ASC, all patients received a face mask and were instructed on how to wear it.

The importance of triage is highlighted by all recommendations from different scientific communities.^14–17^

#### Reduction of droplet generation and infection transmission

As the model of transmission is mainly by droplets, we introduced the following measures:

- The waiting rooms are kept as empty as possible by increasing the interval time between appointments, and as much as prudent, the visits of the most vulnerable patients are reduced.
- We identified a separate, well-ventilated space that allows the waiting patients to be separated by 6 or more feet, with easy access to respiratory hygiene supplies.
- Cough etiquette is promoted.
- Hand hygiene by hand sanitizing with alcoholic solutions is performed. Soap and water are used if hands are visibly soiled. Meticulous hand hygiene is strongly recommended during the following steps: before and after all patient contact; before and after using a surgical mask and after removing gloves; after having contact with respiratory and lacrimal secretions and objects/materials in the environment surrounding the patient.
- We designed and installed disposable slit-lamp plastic barriers as they may provide a measure of added protection against droplet transmission.^18,19^
- Patients and physicians are strongly recommended to avoid speaking during slit-lamp examination.
- Non-contact tonometry (NCT) is a potential source of microaerosol.^20^ Therefore, it is prudent to suspend the use of NCT in outbreak areas. I-Care tonometry or Goldmann applanation tonometry with the use of disposable tips are encouraged to minimize the risk of cross-infection.

An additional preventive action is to reduce time in close contact during examination as illustrated in the key actions by the Royal College of Ophthalmology.^15^

#### Use of personal protective equipment

Healthcare personnel are on the front lines of caring for patients with confirmed or possible infection with COVID-19 and therefore have an increased risk of exposure to this virus. Moreover, it is estimated that a high percentage of healthcare personnel working in outbreak areas are positive for SARS-CoV-2. Eye examinations typically require less than 3-feet face-to-face distance. Therefore, the correct use of personal protective equipment (PPE) is mandatory in order to prevent nosocomial amplification events in ophthalmology.

Theoretically, gown, gloves, protective mask and goggles or some sort of good eye protection are recommended. However, many hospitals in Italy and Europe have reported shortages of PPE, specifically N95 respirators and face masks. In this scenario, alternatives are considered, including other classes of face masks. Special care is taken to ensure that respirators are reserved for situations where respiratory protection is most important, such as during aerosol-generating procedures on suspected or confirmed COVID-19 patients. Physicians and staff wear gloves before touching any patients with conjunctivitis. Attention is paid to training and proper donning, doffing and disposal of any PPE. A recent systematic review^21^ found that frequent hand washing, barrier measures including gloves and masks and isolation of people with suspected respiratory tract infection reduce the transmission of respiratory viruses. Although the protective effect of surgical masks against relevant aerosolized biological hazards is limited, they are still protective to some extent.^22–24^

In Italy, 44% of the laboratory-confirmed cases have been asymptomatic.^25,26^ It has also been reported that some asymptomatic patients have a viral load similar to those with symptoms and that infected persons could transmit infection before symptoms appear.^11,14,26^ Therefore, the use of face masks is reasonable when visiting asymptomatic patients at the slit lamp. Current guidelines of the AAO for urgent ophthalmology appointment for a patient with no respiratory illness symptoms, no fever and no COVID-19 risk factors indicate that masks, gown and gloves are not routinely required for patient or clinician but may reduce asymptomatic transmission. The key actions from the Royal College of Ophthalmologists suggest the use of surgical face masks at discretion during standard slit-lamp examination and the use of surgical face mask in case of prolonged close exam, for example, panretinal photocoagulation (PRP) laser.^15^ Also, local regulations may influence the use of PPE.

#### Environmental control

Rooms and instruments are thoroughly disinfected after each patient encounter. Wear disposable gloves when cleaning and disinfecting surfaces. Slit lamps, including joystick and other controls, and accompanying breath shields, are disinfected. With the use of disposable tips in tonometry, the risk of cross-infection is minimized.^19^ A full list of antimicrobial products expected to be effective against COVID-19 is available online.^27^ Clinical documentation is completed using electronic medical records. Attention is paid to computer mouse and keyboard disinfection. Clinical staff clean workspaces and personal items such as stethoscopes, mobile phones, dictation devices, landlines, name tags and other items with appropriate disinfectants. Environmental service workers increase the frequency of cleaning of commonly touched surfaces such as light switches, countertops, chair arms, elevator buttons, doorknobs and handles. Active decontamination is not merely a technical issue; it is also reassuring to stressed and concerned caregivers, patients and visitors. The importance of environmental control is also highlighted by recent recommendations.^14–17^

#### Staff education, infection control training and symptom monitoring

All staff undergo infection control training to familiarize with the proper steps of hand hygiene and donning and doffing of PPE. All clinical staff are required to report any symptoms such as fever, chills, myalgia, sore throat, runny nose, cough, vomiting, diarrhoea or pneumonia as well as on their recent travel histories. We schedule daily virtual meetings to allow staff to receive updated instructions, and share information and data without being physically grouped together. We utilize a free multi-platform online service.

#### Patient education

Considering the multitude of contacts, it is the responsibility of the staff to provide patient education in order to improve social behaviour and disease understanding:

- Educate to social distancing measures, following Government and Health Authorities’ instructions.
- Educate patients to proper eye drop administration technique. Promote self-instillation.
- Educate eyeglass wearers to disinfect spectacles and glasses. Attention is paid at presbyopic patients using reading glasses, as they may be putting them on and off their face multiple times a day. Promote careful and thorough hand washing.
- Educate contact lens users to proper hand washing before and after insertion and removal.

#### Additional measures to be considered

The following measures are not yet applied to our centres. However, there is a rationale for their adoption in a clinical setting:

- At arrival, all patients and their accompanying persons could be screened using infrared thermometers.
- Electronic messaging services – such as short messaging service (SMS), messaging apps or chatbots – could be used in order to provide prompt and useful information to patients.
- When physical barriers are not present between patients and the admittance staff, alternative measures are adopted such as social distancing (at least 3–6 feet), avoidance of any direct and indirect contact.

## Discussion

As of 30 March 2020, more than 11,000 subjects have been killed in Italy and 37,000 worldwide by COVID-19, which has had the worst outbreak outside of mainland China.^3^ Unlike the Great War, this battle cannot be fought with traditional arms, as very few are known about the enemy and how to fight it. The only weapon that we currently have to avoid the collapse of healthcare services is to reduce the spread of this novel virus through containment measures to mitigate the risk of nosocomial transmission. It is critically important to implement proactive infection control actions, which must be planned ahead. There is a massive risk of paying a high price for the lack of training, appropriate tools and proper plans.

Ophthalmologists are at elevated risk of exposure when caring patients, because they work in close proximity to patients and serve as the first providers to evaluate COVID-19 positive patients with conjunctivitis. Ophthalmologists usually visit a high volume of patients over the age of 60 years, putting them at increased risk of developing COVID-19. Providing patients with a face mask, supplying tissues, promoting cough etiquette and recommending hand hygiene and applying decontamination are all important steps. All healthcare personnel should be aware that they may become a major source of contamination in case they do not attain to simple and yet efficacious hygiene rules. Frequent information and feedback sessions, complemented by clear, concise and measured communication via virtual meetings, will help staff stay focused.

ASCs may react more promptly to pandemic than hospitals due to their lower complexity and confined spaces which enable prompt application of containment measures. During emergency conditions, hospital administrators may consider to reallocate specific procedures such as intravitreal therapies to ASCs in order to limit patients’ access to hospitals, where the risk of being exposed to the infecting agent is higher. We hope that our early experience in enhancing infection control measures in ophthalmology can help physicians to optimally reorganize patient care.
